# Development of a sensitive HPLC‐MS/MS method for 25‐hydroxyvitamin D_2_ and D_3_ measurement in capillary blood

**DOI:** 10.1002/jcla.23451

**Published:** 2020-06-26

**Authors:** XianTing Jiao, Yichun Yuan, Xirui Wang, Juan Li, Bin Liu, Tao Yuan, XiaoDan Yu

**Affiliations:** ^1^ Department of Developmental and Behavioral Pediatrics Pediatric Translation Medicine Institute Shanghai Children’s Medical Center Shanghai Jiao Tong University School of Medicine Shanghai China; ^2^ Department of Pediatric Cardiology Xinhua Hospital Affiliated to Shanghai Jiao Tong University School of Medicine Shanghai China; ^3^ Department of Child Healthcare Shanghai Pudong Yangjing Community Health Service Center Shanghai China; ^4^ College of Food Science Fujian Agriculture and Forestry University Fuzhou China; ^5^ School of Environmental Science and Engineering Shanghai Jiao Tong University Shanghai China

**Keywords:** 25(OH)D, 25(OH)D_3_, capillary blood, high‐performance liquid chromatography‐tandem mass spectrometry

## Abstract

**Background:**

Measurement of 25‐hydroxyvitamin D [25(OH)D)] levels is important. The current method requires a relatively large volume of serum. To minimize the amount of serum needed, we established a high‐performance liquid chromatography‐tandem mass spectrometry (HPLC‐MS/MS) method to measure 25(OH)D in capillary serum.

**Methods:**

Venous blood and fingertip blood were collected from 90 participants. Volumes of 100 µL of venous serum and 20 µL of capillary serum were collected. The serum samples were pretreated by protein removal, extraction and concentration, and an HPLC‐MS/MS method based on chromatographic separation and multi reactive ion monitoring was conducted. The intra‐ and inter‐batch variation coefficients were less than 10% for both 25‐hydroxyvitamin D_3_[25(OH)D_3_] and 25‐hydroxyvitamin D_2_[25(OH)D_2_)]. For venous specimens, the accuracies were 3.87% and 4.91%, respectively. For capillary specimens, the accuracies were 1.65% and 5.32%, respectively.

**Results:**

The limit of detection (LOD) of 25(OH)D_3_ was 0.01 ng/mL, and the LOD of 25(OH)D_2_ was 0.05 ng/mL. The results showed that the mean concentration of 25(OH)D in venous blood was 22.56 ± 9.50 ng/mL, while the mean concentration of 25(OH)D in capillary blood was 18.14 ± 7.86 ng/mL. Furthermore, the adjusted capillary blood 25(OH)D level was 22.99 ± 10.24 ng/mL by the correction formula in our study. Similarly, the mean concentration of 25(OH)D_3_ in capillary blood was 17.98 ± 7.98 ng/mL. The adjusted capillary blood 25(OH)D_3_ level was 22.85 ± 10.42 ng/mL. No difference in the content of 25(OH)D or 25(OH)D_3_ was found between venous serum and corrected capillary serum. The correlation coefficients between venous and corrected capillary concentrations of 25(OH)D and 25(OH)D_3_ were 0.7941 and 0.8103, respectively, and the areas under the receiver operating characteristic curve were 0.9367 and 0.9565, respectively.

**Conclusions:**

This capillary blood method requires minimal sample preparation and is suitable for routine use in the 25(OH)D detection.

## Graphical abstract

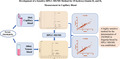

## 1 INTRODUCTION

Vitamin D is a fat‐soluble vitamin that functions as a steroid hormone. It plays a crucial role in mineral homeostasis and skeletal health.^1^ Vitamin D deficiency is increasing in the general population and is considered an important public health problem with serious effects.^2^, ^3^ It is necessary to implement widespread vitamin D testing among the public. In particular, key populations should be screened for vitamin D deficiency or insufficiency.

25‐hydroxyvitamin D [25(OH)D] is the main circulating form of vitamin D in serum, which includes 25 (OH) D_3_ and 25 (OH) D_2_, and has been measured by various methods as a reliable clinical indicator of vitamin D status.^4^ Four basic methods are currently available for the determination of 25(OH)D: (1) The competitive protein binding test (CPBA), (2) the competitive immune test, (3) liquid chromatography‐tandem mass spectrometry (LC‐MS/MS),^5^, ^6^ and (4) high‐performance liquid chromatography (HPLC). To data, high‐performance liquid chromatography‐tandem mass spectrometry (HPLC‐MS/MS) assays in clinical laboratories have exhibited better sensitivity, accuracy, and reproducibility than older methods for detecting 25(OH)D metabolites.^7^, ^8^ Because of these advantages, HPLC‐MS/MS is often known as the “gold standard” method for the measurement of 25(OH)D metabolites.^9^ Since 2008, HPLC‐MS/MS has been the method recommended by the National Health and Nutritional Examination Survey (NHANES) for quantifying 25(OH)D.^10^ The NHANES investigated the measurement error of 25(OH)D analysis by immunoassays and suggested replacing the DiaSorin radioimmunoassay with HPLC‐MS/MS in the future.^11^ Furthermore, the UK Food Standards Agency (FSA) instructed scientists to use HPLC‐MS/MS over other methods as part of the National Diet and Nutrition Survey (NDNS).^12^

However, the current classical method of HPLC‐MS/MS requires a relatively large quantity of serum samples. Additionally, conventional arterial or venous blood collection methods are invasive and could potentially cause pain, needle stick injuries, and contamination.^13^ In particular, it is difficult to extract venous blood from children. Thus, it is urgent to develop a method that requires only trace blood to detect. At present, blood glucose,^14^ viruses, blood lead,^15^ and other analytes can be detected from microblood in routine medical practice. Capillary blood analysis is relatively easy to perform and requires only a small volume of blood (<100 μL) obtained via a finger stick. Second, the test is much less costly than a venous blood test. Third, there are no environmental contamination problems associated with the Eppendorf (EP) tubes, even when tests are performed with small capillary blood specimens.^16^

To address the shortcomings of venous blood testing, this study gathered 90 matched pairs of capillary and venous blood specimens collected on the same day during routine visits to a pediatric healthcare clinic and used them to establish a new HPLC‐MS/MS method for quantitative determination of 25(OH)D in capillary blood.

## 2 MATERIALS AND METHODS

### 2.1 Chemicals

25(OH)D_2_ (100 µg/mL, purity 98%), 25(OH)D_3_ (100 µg/mL, purity 98%), 25(OH)D_2_‐d_3_ (internal standard [IS] for 25(OH)D_2_, 100 µg/mL, purity 98%), 25(OH)D_3_‐d_6_ (IS for 25(OH)D_3_, 0.5 mg, purity 95%), and formic acid were purchased from Sigma‐Aldrich (St. Louis, MO, USA). Standard reference materials SRM 2972 and SRM 972a were purchased from the National Institute of Standards and Technology (NIST). Organic solvents, hexane and methanol (HPLC grade), were obtained from Fisher Scientific (Pittsburgh, PA, USA). ZnSO_4_ was purchased from China National Medicines Corporation Ltd (Beijing, China).

### 2.2 Preparation of calibrators

Mixed calibration standards were prepared at concentrations of 6.25/6.25, 12.5/12.5, 25/25, 50/50, 125/125, 250/250, and 500/500 nmol/L (25(OH)D_2_/25(OH)D_3_) in methanol. The standards were certified according to NIST SRM2972. All calibrators were treated as samples in each batch.

### 2.3 Serum samples

From March to August 2018, venous blood and fingertip blood were collected simultaneously from 90 participants who came from the Department of Developmental and Behavioral Pediatrics, Shanghai Children's Medical Center, Shanghai Jiao Tong University School of Medicine for 25(OH)D tests. Venous blood samples were taken by venipuncture into tubes containing a clot activator. The blood collected from the fingers was squeezed into an EP tube containing the anticoagulant ethylene diamine tetraacetic acid (EDTA). The serum was isolated, stored at − 20°C in the dark, and then analyzed using both HPLC‐MS/MS methods. The present study was conducted according to the guidelines established in the Declaration of Helsinki. This study protocol was approved by the medical ethics committee of Shanghai Children's Medical Center affiliated with Shanghai Jiao Tong University School of Medicine, and all study participants provided written informed consent. Ethics number: SCMCIRB‐K2018061.

### 2.4 Detection analysis of venous serum 25(OH)D

The HPLC‐MS/MS methods in this work were carried out on a Shimadzu 8040 detection instrument (Shimadzu Company, Japan). A volume of 100 µL of venous serum was collected and extracted, and the serum samples were pretreated by protein removal, extraction, and concentration. The chromatograms of 25(OH)D_2_ and 25(OH)D_3_ were obtained by liquid chromatography and then analyzed by mass spectrometry. The Shimadzu 8040 mass spectrometer was equipped with Electrospray ionization (ESI) and operated in the positive ionization mode. The precursor ions (m/z 401.3 for 25OHD_3_, m/z 413.3 for 25OHD_2_, and m/z 407.3 for 25(OH)D_3_‐d_6_, m/z 416.3 for 25(OH)D_2_‐d_3_) was selected by the multi‐reaction ion monitoring (MRM) approach and then subsequently fragmented to produce specific product ions for data analysis (Table 1). Mass spectrometer settings were as follows: atomizer temperature 350℃, atomizing gas flow 6 L/min, atomizing air pressure 35 psi, DL tube temperature 150℃.The standard curve equation was determined from the data of the instrument test standard substances (Sigma standard substances). The relative standard deviation was less than 15%. According to the ratio of the peak area of vitamin D_2_ (or vitamin D_3_) to the peak area of the IS substance, the contents of 25(OH)D_3_ and 25(OH)D_2_ in quality control samples and samples to be tested were calculated according to the standard curve equation.

**TABLE 1:** Reaction monitoring acquisition settings parameters of venous blood based on HPLC‐MS/MS method

Analyte	25(OH)D_3_	25(OH)D_3_‐d_6_	25(OH)D_2_	25(OH)D_2_‐d_3_
Precursor ion (m/z)	401.3	407.3	413.3	416.3
Product ion (m/z)	365.3	371.3	337.3	340.3
Dwell Time（msec）	100	100	100	100
Collision energy（volts）	17	17	16	16

The quality control range was ‾x ± 2s. The Westgard multirule quality control method was used to determine whether the quality of the results was acceptable. At least two quality control measurements were made in each batch of samples. The intra‐ and inter‐batch variation coefficients were less than 5% for both 25(OH)D_3_ and 25(OH)D_2_.

### 2.5 Detection analysis of capillary serum 25(OH)D

The detection instrument was a high‐performance liquid chromatography‐tandem mass spectrometry apparatus (AB Sciex 4500MD, AB Sciex Company, USA). A 20 µL volume of capillary serum was collected, and the capillary serum was pretreated by protein removal, extraction, and concentration. The chromatograms of 25(OH)D_2_ and 25(OH)D_3_ were obtained by liquid chromatography and then analyzed by mass spectrometry, which condition is the key to detect 25 (OH)D concentration in capillary serum. The AB Sciex 4500MD mass spectrometer was equipped with electrospray ionization (ESI) and operated in the positive ionization mode. The precursor ions (m/z 401.3 for 25OHD_3_, m/z 413.3 for 25OHD_2_, and m/z 407.3 for 25(OH)D_3_‐d_6_, m/z 416.3 for 25(OH)D_2_‐d_3_) were selected by the multi‐reaction ion monitoring (MRM) approach and then subsequently fragmented to produce specific product ions for data analysis (Table 2). Mass spectrometer settings were as follows: The temperature of the ion source is 400℃, the spray pressure is 70 psi, the air curtain pressure is 20 psi, and the ionization voltage is 4500 V.

**TABLE 2:** Reaction monitoring acquisition settings parameters of capillary blood based on HPLC‐MS/MS method

Analyte	25(OH)D_3_	25(OH)D_3_‐d_6_	25(OH)D_2_	25(OH)D_2_‐d_3_
Precursor ion (m/z)	401.3	407.3	413.3	416.3
Product ion (m/z)	365.3	371.3	337.3	340.3
Dwell Time（msec）	100	100	100	100
Collision energy（volts）	17	17	15	15

The relative standard deviation was less than 15%. The content of 25(OH)D_3_ and 25(OH)D_2_ in quality control samples and samples to be tested was calculated according to the standard curve equation. The quality control range was ‾x ± 2s. The Westgard multirule quality control method was used to determine whether the quality of the results was acceptable. At least two quality control measurements were made from each batch of samples. The intra‐ and inter‐batch variation coefficients were less than 10% for both 25(OH)D_3_ and 25(OH)D_2_.

### 2.6 Method comparison

The two HPLC‐MS/MS methods were applied to 90 serum samples from patients (36 boys and 54 girls aged 3‐10 years) who requested a 25(OH)D test in our clinical laboratory. Bland‐Altman plots were used to identify the mean bias (the average of the difference between the measurements obtained from the two assays was also compared), and a 95% limit of agreement was observed between methods. Agreement between the assays regarding 25(OH)D status was assessed using Cohen's kappa (agreement: <0.4, poor; 0.4‐0.75, fair to good; >0.75, excellent). *P*<.05 was considered statistically significant. All statistical analyses were performed using EmpowerStats and GraphPad statistical software.

## 3 RESULTS

### 3.1 Analytical performance evaluation of the HPLC‐MS/MS methodology

**FIGURE 1:**
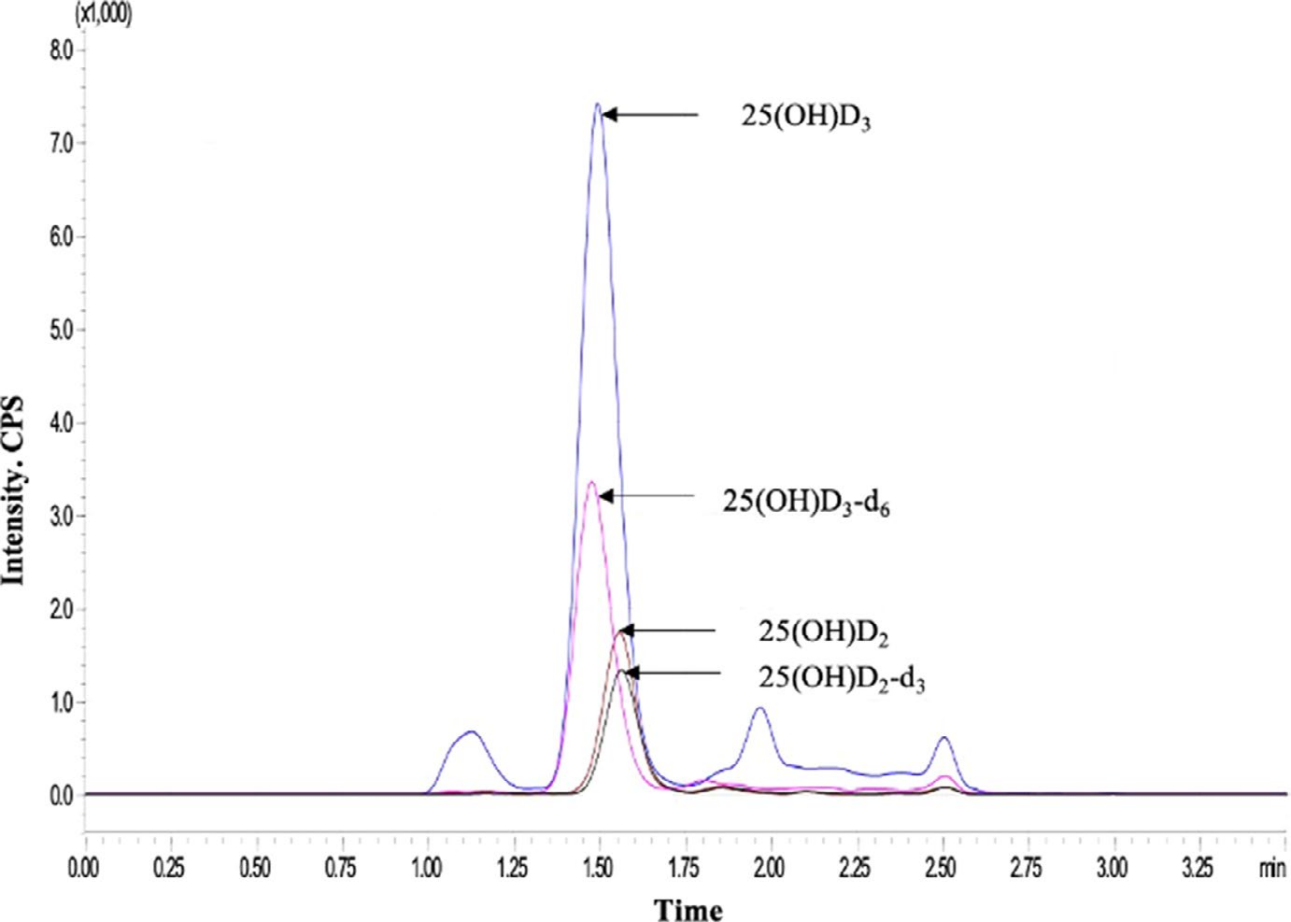
Representative MRM chromatograms of a venous serum specimen

The HPLC‐MS/MS chromatograms of a venous sample are shown in Figure 1. Using the HPLC‐MS/MS procedure, the vitamin D metabolites and IS were adequately separated, with 25(OH)D_3_ eluting at 1.50 minute and its IS eluting at 1.45 min, while 25(OH)D_2_ eluted at 1.60 minute and its IS eluted at 1.61 minute. Potential interfering substances such as 3‐epi‐25(OH)D were eluted between 1.00 and 1.25 minute or 2.00 minute but did not interfere with the detection of 25(OH)D_2_ and 25(OH)D_3_.

**FIGURE 2:**
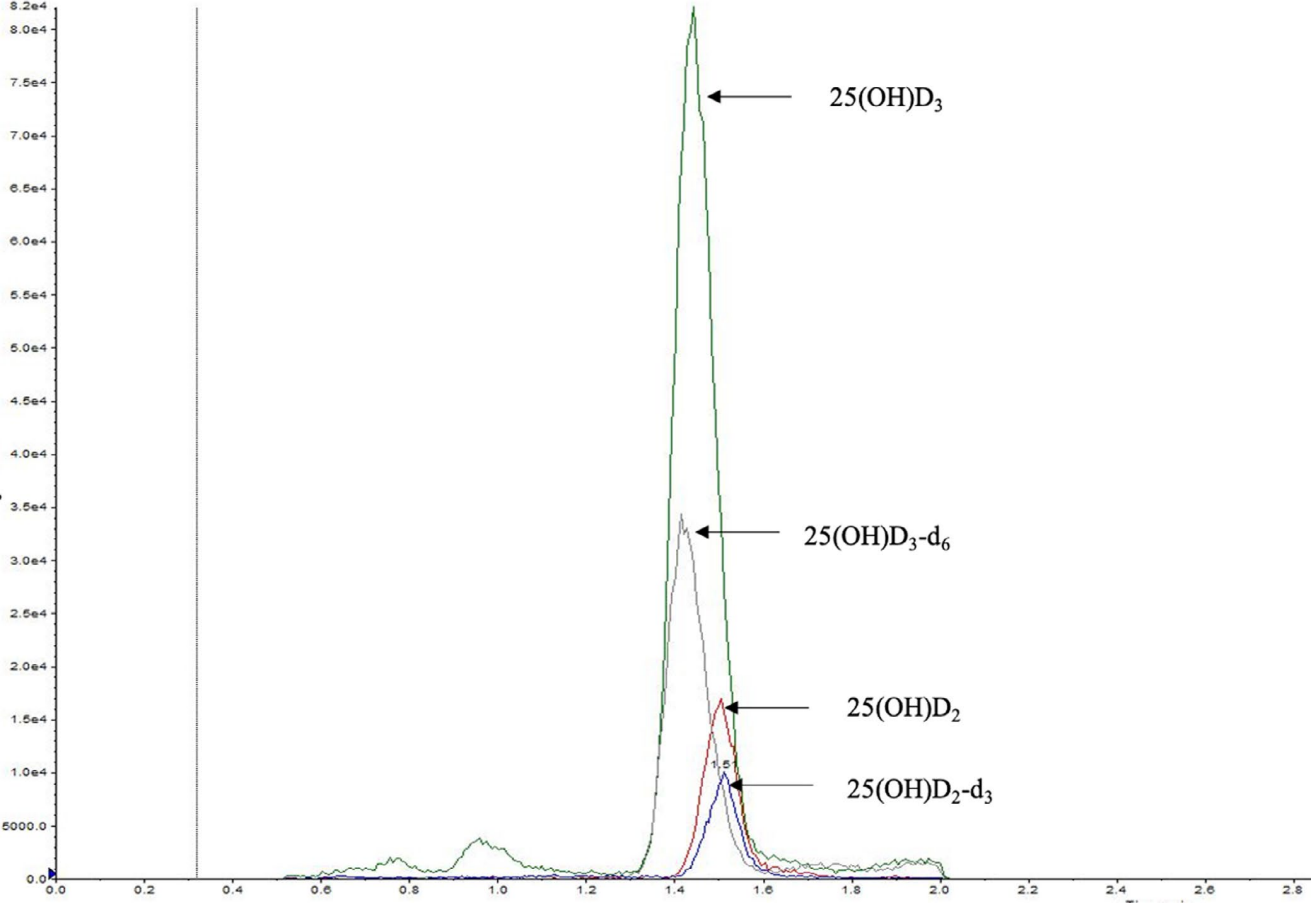
Representative MRM chromatograms of a capillary serum specimen

The HPLC‐MS/MS chromatograms of a capillary sample are shown in Figure 2. Using HPLC‐MS/MS procedure, the vitamin D metabolites and IS were adequately separated, with 25(OH)D_3_ eluting at 1.40 minute and its IS eluting also at 1.40 minute, while 25(OH)D_2_ eluted at 1.50 minute and its IS eluted at 1.50 minute. Potential interfering substances such as 3‐epi‐25(OH)D were eluted at 1.00 minute or 2.00 minute but did not interfere with the detection of 25(OH)D_2_ and 25(OH)D_3_.

### 3.2 Validation results of the HPLC‐MS/MS assay method of venous and capillary specimens

For venous specimens, the limit of detection (LOD) of 25(OH)D_3_ was 0.01 ng/mL, and the LOD of 25(OH)D_2_ was 0.05 ng/mL. The limit of quantification (LOQ) of 25(OH)D_3_ was 1.5 ng/mL, and the LOQ of 25(OH)D_2_ was 1.5 ng/mL. When 25(OH)D_2_ is in the range of 0.8 ng/mL to 50.0 ng/mL, the linearity is good, and the correlation coefficient *R* ^2^> 0.99. When 25(OH)D_3_ is in the range of 2.5 ng/mL to 160.0 ng/mL, the linearity is good, and the correlation coefficient *R* ^2^> 0.99. In the determination of 25(OH)D_3_, three concentrations of 10 ng/mL, 40 ng/mL, and 160 ng/mL were set for quality control. In the determination of 25 (OH) D_2_, three concentrations of 5, 10, and 40 ng/mL were set for quality control. The recovery (%) of the accuracy of the two compounds was in a reasonable range of 85%~115%. The accuracies were 3.87% and 4.91%, respectively (Table 3).

**TABLE 3:** Validation results of the HPLC‐MS/MS assay method of venous specimens

		Low concentration	Medium concentration	High concentration
Additional Standard Matter Amount (ng/mL)	25(OH)D_3_	10	40	160
	25(OH)D_2_	5	10	40
Recovery (%)	25(OH)D_3_	91.37	103.51	97.43
	25(OH)D_2_	100.21	102.52	100.96
Accuracy (%)	25(OH)D_3_	3.87		
	25(OH)D_2_	4.91		

For capillary specimens, the LOD of 25(OH)D_3_ was 0.01 ng/mL, and the LOD of 25(OH)D_2_ was 0.05 ng/mL. The LOQ of 25(OH)D_3_ was 5 ng/mL, and the LOQ of 25(OH)D_2_ was 0.8 ng/mL. When 25(OH)D_2_ is in the range of 0.10 ng/mL to 12.50 ng/mL, the linearity is good, and the correlation coefficient *R* ^2^> 0.99. When 25(OH)D_3_ is in the range of 0.31 ng/mL to 40.0 ng/mL, the linearity is good, and the correlation coefficient *R* ^2^> 0.99. In the determination of 25(OH)D_3_, three concentrations of 1.25 ng/mL, 5.00 ng/mL, and 20.00 ng/mL were set for quality control. In the determination of 25 (OH) D_2_, three concentrations of 0.39, 1.56, and 6.25 ng/mL were set for quality control. The recovery (%) of the accuracy of the two compounds was in a reasonable range of 85%~115%. The accuracies were 1.65% and 5.32%, respectively (Table 4).

**TABLE 4:** Validation results of the HPLC‐MS/MS assay method of capillary specimens

		Low concentration	Medium concentration	High concentration
Additional Standard Matter Amount (ng/mL)	25(OH)D_3_	1.25	5	20
	25(OH)D_2_	0.39	1.56	6.25
Recoveries (%)	25(OH)D_3_	98.52	100.81	105.54
	25(OH)D_2_	103.89	96	97.16
Accuracy (%)	25(OH)D_3_	1.65		
	25(OH)D_2_	5.32		

### 3.3 25(OH)D levels in venous and capillary specimens

The results of 25(OH)D concentration in venous and fingertip blood of the three groups were compared. The quantity of 25(OH)D equals the sum of the quantities of 25(OH)D_3_ and 25(OH)D_2_. Differences in the mean values of 25(OH)D and 25(OH)D_3_ in venous blood and capillary blood were found among the three groups (Table 5). Since 92% of samples contained less than 0.1 ng/mL 25(OH)D_2_, we did not include that analyte in the subsequent statistical analyses.

**TABLE 5:** Comparison of HPLC‐MS/MS 25(OH)D/25(OH)D_3_ measurements and precision between venous and capillary specimens

	Group	Venous serum			Capillary serum			*P*	LOD
		n	Mean	SD	n	Mean	SD		
25(OH)D (ng/ml)	Group 1	30.0	26.58	9.34	30	18.52	6.96	＜.001*	0.01
	Group 2	30.0	20.56	8.42	30	18.88	7.63	.01*	0.01
	Group 3	30.0	20.53	9.68	30	17.68	8.54	＜.001*	0.01
	Total	90.0	22.56	9.50	90	18.14	7.86	.001*	
25(OH)D_3_ (ng/ml)	Group 1	30.0	25.91	9.89	30	18.12	7.37	＜.001*	0.05
	Group 2	30.0	20.56	8.39	30	18.32	7.52	.02*	0.05
	Group 3	30.0	20.07	9.73	30	17.29	8.58	＜.001*	0.05
	Total	90.0	22.18	9.64	90	17.98	7.98	.002*	

* *P* < .05.

### 3.4 Comparisons of the results of two specimens

**FIGURE 3:**
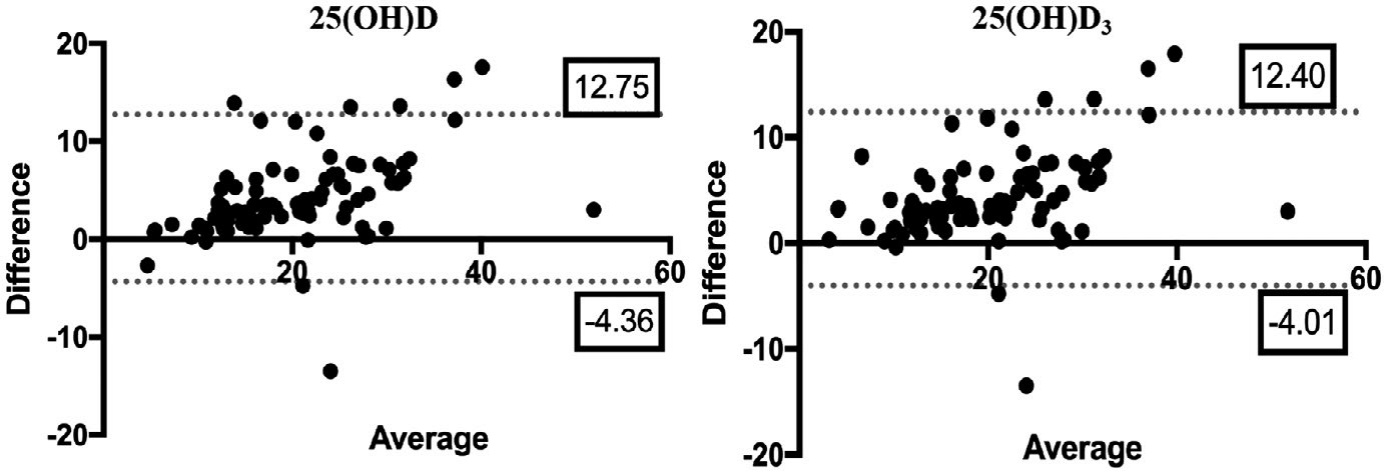
Bland‐Altman consistency test scatter plots of two specimens

Furthermore, we assessed the consistency of the results from the two groups using the Bland‐Altman method (Figure 3). The 95% consistency limit for 25(OH)D was (−4.36, 12.75). The bias between the two samples was 4.196, and the standard deviation (SD) of the bias was 4.365, which indicated that the 25(OH)D results from the two samples were in good agreement. Similarly, the 95% consistency limit was (−4.01, 12.40) of 25(OH)D_3_. We constructed the following model for converting between the two types of specimens: log(corrected capillary 25(OH)D) = 0.01049 + 1.06692 * log(capillary 25(OH)D), log(corrected capillary 25(OH)D_3_) = 0.02864 + 1.05947 * log(capillary 25(OH)D_3_). The capillary 25(OH)D and 25(OH)D_3_ measurements were transformed according to the formula.

### 3.5 Comparing the results of 25(OH)D and 25(OH)D_3_ after correction

**FIGURE 4:**
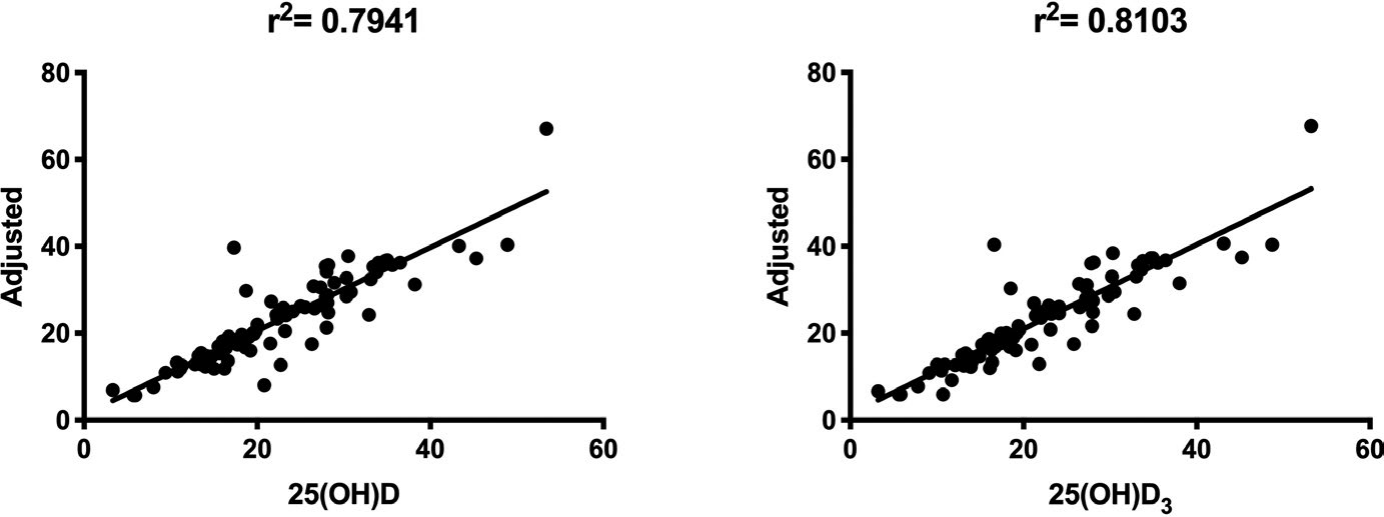
Correlation analysis between venous 25(OH)D/25(OH)D_3_ and adjusted capillary 25(OH)D/25(OH)D_3_

**FIGURE 5:**
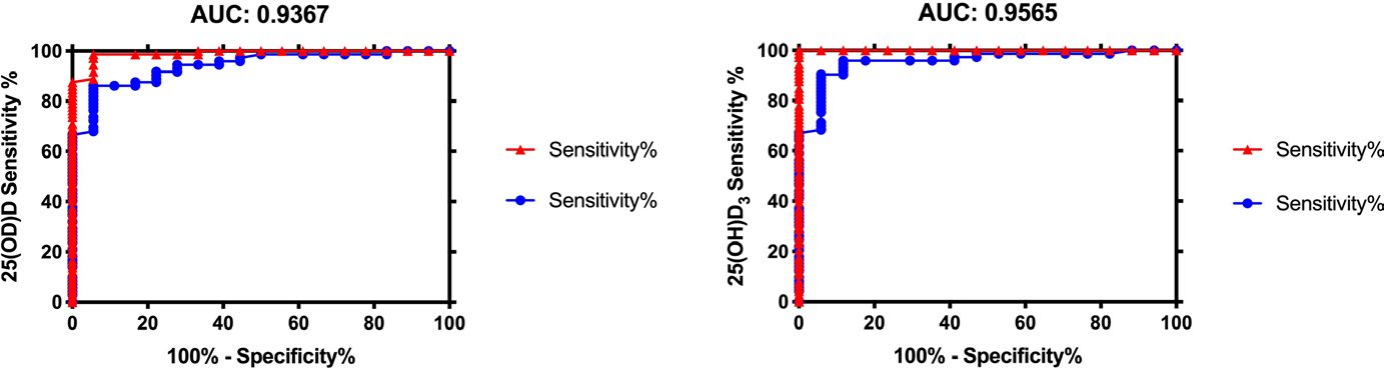
ROC curve analysis of venous 25(OH)D/25(OH)D_3_ and adjusted capillary 25(OH)D/25(OH)D_3_

There was no difference in the mean concentration of 25(OH)D or 25(OH)D_3_ between venous blood and corrected capillary blood among the three groups (Table 6). Accordingly, the data from the three groups were pooled. The correlation between the venous and corrected capillary measurements for 25(OH)D and 25(OH)D_3_ had a coefficient of 0.7941 and 0.8103, respectively (Figure 4), and the AUC was 0.9367 and 0.9565, respectively (Figure 5). The analysis of the above results demonstrates that the detection of 25(OH)D and 25(OH)D_3_ in peripheral blood by HPLC‐MS/MS has high accuracy and sensitivity.

**TABLE 6:** Comparison of venous blood 25(OH)D/25(OH)D_3_ and corrected capillary blood 25(OH)D/25(OH)D_3_ results

	Group	Venous serum			Corrected capillary serum			*P*
		n	Mean	SD	n	Mean	SD	
25(OH)D (ng/ml)	Group 1	30.0	26.58	9.34	30	23.17	9.26	.65
	Group 2	30.0	20.56	8.42	30	23.69	10.10	.46
	Group 3	30.0	20.53	9.68	30	22.11	11.53	.54
	Total	90.0	22.56	9.50	90	22.99	10.24	.77
25(OH)D_3_ (ng/ml)	Group 1	30.0	25.91	9.89	30	20.56	8.42	.27
	Group 2	30.0	20.56	8.39	30	23.69	10.10	.14
	Group 3	30.0	20.07	9.73	30	19.81	8.30	.48
	Total	90.0	22.18	9.64	90	22.85	10.42	.53

## 4 DISCUSSION

In this study, based on the classical method for determination of 25(OH)D in venous serum by HPLC‐MS/MS, we established a new method for determination of 25(OH)D in finger stick blood by HPLC‐MS/MS. The method is a simple, rapid process with high sensitivity, few opportunities for interference, and good accuracy and reproducibility. In short, these advances make capillary blood a promising sample source for 25(OH)D screening.

To develop the practical application value of this method, we selected 90 child volunteers and measured the content of 25(OH)D in their venous blood and capillary blood simultaneously. The results showed that the mean concentration of 25(OH)D in venous blood was 22.56 ± 9.50 ng/mL, which is consistent with values reported in the literature.^17^ The mean concentration of 25(OH)D in fingertip blood was 17.98 ± 7.98 ng/mL, which was slightly lower than that in venous blood. This result was not surprising in light of a previous study that compared the concentrations of 12 chemical constituents between capillary serum or plasma and venous serum.^18^ That study concluded that there were differences in the concentrations of potassium, total protein, and calcium between venous serum and capillary serum or plasma. The probable explanation for the lower concentrations in skin‐puncture blood specimens is that they are mixed with interstitial fluid. These differences should be considered when comparing these types of specimens. Therefore, in our study, we corrected the capillary 25(OH)D results using the correction formula. The adjusted capillary blood 25(OH)D level was 22.99 ± 10.24 ng/mL, which showed that corrected capillary blood yields results for 25(OH)D that are not significantly different from the corresponding values measured in venous blood. The correlation coefficient between the capillary 25(OH)D value and the venous value was 0.7941. These results indicate that 25(OH)D levels in capillary blood are a good reflection of the amounts in venous samples.

For 25(OH)D_3_, the lower limit of this detection for this method is 0.01 ng/mL, which is better than the reported value for the existing method. In contrast to the current reliable LC‐MS/MS method for detection of 25(OH)D_2_ and 25(OH)D_3_ in venous serum,^19^, ^20^, ^21^ the advantage of this study is that 25(OH)D was detected in micro blood by an HPLC‐MS/MS method. The detection instrument was a point‐of‐care HPLC‐MS/MS apparatus for molecular diagnostics using a drop of blood (AB Sciex 4500MD, AB Sciex Company, USA). In contrast to the complex pretreatment methods required for most tests, ours requires only the collection of a 20 µL finger blood sample. After the protein and impurities were removed, the active ingredients were further extracted. Then, chromatographic separation and mass spectrometry were used for MRM ion monitoring, and the IS method was used for quantitative analysis. The detection of 25(OH)D in capillary blood by HPLC‐MS/MS is the main feature of this study. Importantly, compared to arterial and venous blood, capillary blood (such as the samples used in this study) can be easily collected from a patient's finger with simple instruments, and the collection method is a simple, rapid, and inexpensive process that does not require a skilled worker. Therefore, capillary blood has excellent potential as an ideal sample source for 25(OH)D screening and other health monitoring.

This study has several limitations. First, the sample sizes were small, with a study population of only 90 volunteers. Nonetheless, the intra‐ and inter‐batch variation coefficients were less than 5% for both 25(OH)D_3_ and 25(OH)D_2_. Hence, this study demonstrates that an HPLC‐MS/MS technique such as ours would be effective for screening young children in the field. Second, data on some important confounders, such as diet and 25(OH)D supplementation, were unavailable. However, considering the sensitivity of this method, the influence of diet and 25(OH)D supplementation should be limited. Third, the retest validity and intertester reliability of our 25(OH)D assessment were not evaluated, which may obscure the measurement error.

## 5 CONCLUSION

A highly sensitive method for the determination of 25(OH)D in fingertip blood by HPLC‐MS/MS was established. The method has few interfering factors, and the results are accurate and reliable. It is suitable for large‐scale determination of 25(OH)D status for population‐wide nutrition surveys. In the context of a public health program to screen 25(OH)D status, the finger preparation protocol would add convenience due to the reduced cost, shortened collection times, and increased safety for collection‐site personnel.

## CONFLICT OF INTEREST

The authors have no financial or personal conflicts of interest to declare.

## AUTHORS' CONTRIBUTIONS

XJ contributed significantly to the analysis and wrote the manuscript; YY conceived and performed the experiments; XW and JL helped perform the analysis; BL and TY participated in constructive discussions; and XY designed the experiments and approved the final version.
